# Taxonomic study of the genus *Meleonoma* Meyrick from Thailand (Lepidoptera, Gelechioidea)

**DOI:** 10.3897/zookeys.571.6897

**Published:** 2016-03-07

**Authors:** Aihui Yin, Shuxia Wang

**Affiliations:** 1 College of Life Sciences, Nankai University, Tianjin 300071, China

**Keywords:** Lepidoptera, Oecophoridae, *Meleonoma*, new species, Thailand

## Abstract

Five species of the genus *Meleonoma* Meyrick are reported from Thailand. *Meleonoma triangula* Wang, **sp. n.**, *Meleonoma dorsolobulata* Wang, **sp. n.**, *Meleonoma elongata* Wang, **sp. n.**, and *Meleonoma bilobata* Wang, **sp. n.** are described as new; *Meleonoma facialis* Li & Wang, 2002 is redescribed and recorded for the first time from Thailand.

## Introduction

Meyrick (1914) established the genus *Meleonoma* in the family Oecophoridae, with *Cryptolechia stomota* Meyrick, 1910 as the type species. Gaede (1939) listed eleven *Meleonoma* species: three from Australia, two from India and two Sri Lanka, and one each from Madagascar, China, Sikkim and Borneo. Common (1996) synonymized *Meleonoma basanista* Meyrick, 1922 with *Oresitropha pentochra* (Lower, 1894), and placed *Meleonoma psammota* Meyrick, 1915 in the subfamily Oecophorinae; Edwards and Nielsen (1996) placed *Meleonoma capnodyta* (Meyrick, 1906), earlier transferred from *Borkhausenia*, in the family Cosmopterigidae. Viette (1955) recognized one species from Madagascar. Clarke (1965) transferred *Pseudodoxia crocomitra* Meyrick, 1914 to *Meleonoma* and placed *Meleonoma* in the family Cosmopterigidae. Subsequently, Li and Wang (2002, 2004) described five species from China, and treated *Meleonoma* as a member of Cosmopterigidae. Lvovsky (2015) described five new *Meleonoma* species from Nepal and China; in the same paper, Lvovsky synonymized *Acryptolechia* that he established in 2010 in the family Cryptolechiidae with *Meleonoma*, and placed *Meleonoma* in the family Lypusidae. In a more recent study the Cryptolechiinae were recognized as a subfamily of the enlarged Depressariidae (Heikkila et al. 2014), but *Meleonoma* was not included in that study.

The taxonomic positions and validity of *Acryptolechia* and *Meleonoma* needs to be further studied and confirmed since they have been reassigned so frequently. A combination of both molecular and morphological analyses is likely a method to resolve such a taxonomic problem, which is not the aim of the present study. We therefore treat *Meleonoma* as an unplaced genus in Gelechioidea. The present paper is to report the result of our recent study of the genus *Meleonoma* in Thailand, including descriptions of four new species.

## Material and methods

The examined specimens were collected from Thailand in 1984, and were borrowed from the Natural History Museum of Denmark, where all types are deposited. Genitalia dissection and mounting methods follow the methods introduced by Li (2002). Photographs of adults were taken with a Leica M205A stereomicroscope plus Leica Application Suite 4.2 software, and illustrations of genitalia were prepared using a Leica DM750 microscope.

## Taxonomy

### Genus Meleonoma

Taxon classificationAnimaliaLepidopteraCosmopterigidae

Meyrick, 1914

Meleonoma Meyrick, 1914: 255.

#### Type species.

*Cryptolechia stomota* Meyrick, 1910, by original designation.

#### Diagnosis.

The genus *Meleonoma* is characterized by the narrow lanceolate forewing with ground color pale yellow, yellow or pale ochreous yellow; by the male genitalia with spear-shaped or slender sticklike uncus, the absent, or membranous or weakly sclerotized circular gnathos, the varied shape of the sacculus, and the elongate or triangular saccus; by the female genitalia with entirely or partly sclerotized ductus bursae, and the signum often with spines, if present.

### Meleonoma triangula

Taxon classificationAnimaliaLepidopteraCosmopterigidae

Wang sp. n.

http://zoobank.org/817C3454-64F0-459E-B972-0FF370C37CB5

Figs 1 , 6 , 11

#### Type material.

**Holotype**, ♂, **Thailand**: Nakhon Nayok Prov., Khao Yai Nat. Park, ca. 700 m, 29.ix.–6.x.1984, leg. Karsholt, Lomholdt & Nielsen, genitalia slide No. ZMUC-NK037. **Paratypes**: 1♀, 1♂, Loei Prov. Phu Luang Wildlife Sanctuary, ca. 700–900 m, 8–14.x.1984, leg. Karsholt, Lomholdt & Nielsen.

#### Diagnosis.

This new species can be distinguished from its congeners by the distal half of the valva distinctly triangular and upturned, and the short sacculus wider than long in the male genitalia.

#### Description.

**Figures 1–5.**
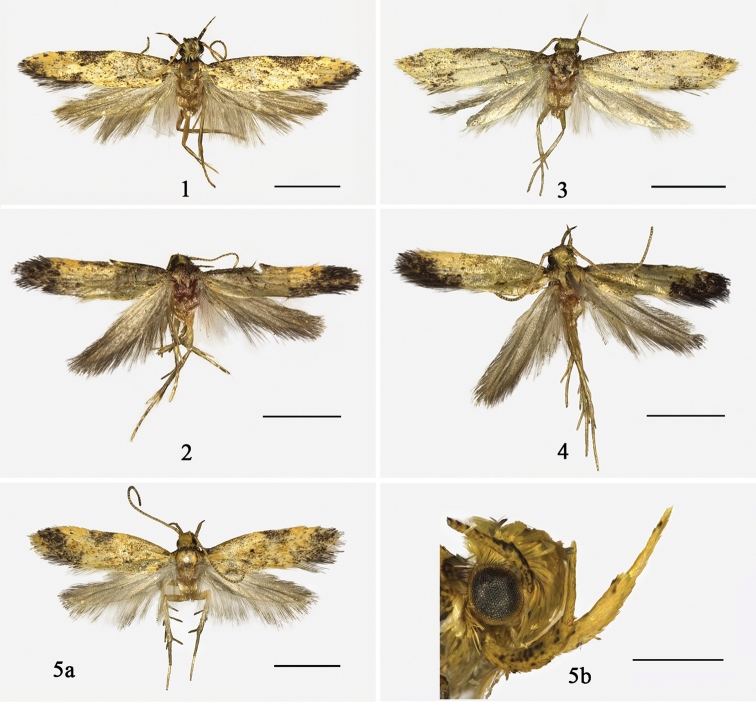
Adults of *Meleonoma* spp. **1** *Meleonoma triangula* sp. n., holotype, male **2** *Meleonoma dorsolobulata* sp. n., holotype, male **3** *Meleonoma elongata* sp. n., holotype, male **4** *Meleonoma bilobata* sp. n., holotype, male **5a** *Meleonoma facialis* Li & Wang, 2002, male **5b** Head of *Meleonoma facialis* Li & Wang, 2002. Scale bars: 2.0 mm (**1−5a**), 500 um (**5b**).

Adult (Fig. 1): Wing expanse 10.0–12.0 mm. Head pale yellow, with scales copper brown tipped. Labial palpus pale yellow, covered with dense copper brown scales on entire second segment and on distal half of third segment. Antenna pale yellow, with dorsal surface brown on scape, ringed with pale brown on flagellum. Thorax yellowish brown; tegula greyish brown mottled yellow. Forewing yellow, with blackish brown scales throughout, concentrated along costal and ventral margins and at apex, forming blackish blotch or spots; costal margin with a large ill-defined inverted triangular blotch beyond middle, and with three small spots along distal 1/6; small black spot at base, middle and end of cell as well as at 2/3 of fold, respectively; cilia greyish brown. Hindwing and cilia pale grey. Legs brown on fore and mid tibiae and all tarsi except pale yellow at apex of each segment; hind leg brown on outer surface except pale yellow at apex.

**Figures 6–11.**
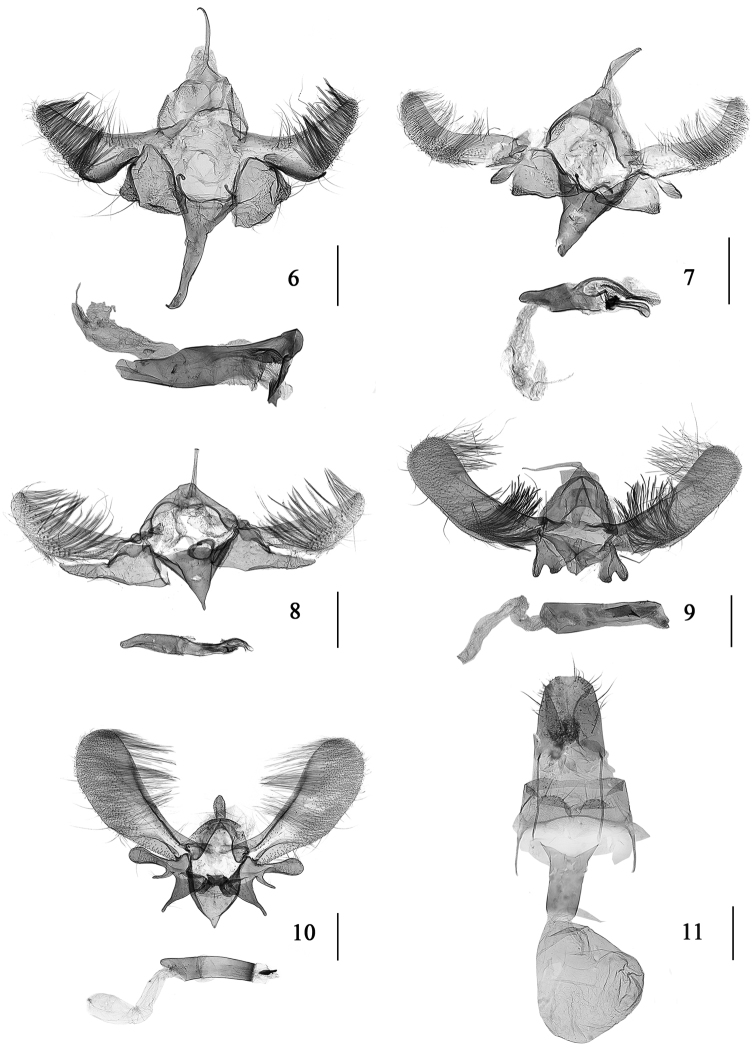
Male genitalia of *Meleonoma* spp. **6** *Meleonoma triangula* sp. n., holotype, slide no. ZMUC-NK037 **7** *Meleonoma dorsolobulata* sp. n., holotype, slide no. ZMUC-NK047 **8** *Meleonoma elongata* sp. n., holotype, slide no. ZMUC-NK053 **9** *Meleonoma bilobata* sp. n., holotype, slide No. ZMUC-NK046 **10** *Meleonoma facialis* Li & Wang, 2002, slide no. ZMUC-NK054 **11** Female genitalia of *Meleonoma triangula* sp. n., paratype, slide no. ZMUC-NK038. Scale bar = 0.25 mm.

Male genitalia (Fig. 6): Uncus twice as long as middle height of tegumen, slender, hooked distally. Gnathos weakly sclerotized. Valva narrow at base, gradually widened to approximately middle; distal half triangular, upturned, obviously narrowed to rounded apex; costa concave shallowly; ventral margin with a sclerotized narrow edge extending from near base to middle length, where it is produced to a small process exceeding ventral margin, with long dense setae on distal half; transtilla small, triangular, pointed distally. Sacculus subrectangular, length shorter than width, apically produced to a hairy papillary process. Saccus longer than uncus, narrowed slightly to apex. Juxta a thin band in semicircular shape. Aedeagus slightly longer than valva, blunt apically, with dense microspines on inner surface in distal third, with an irregular narrow plate before apex.

Female genitalia (Fig. 11): Papillae anales large and broad, setose. Posterior apophyses twice as long as anterior apophyses. Eighth tergite sclerotized posteriorly; eighth sternite with granules posteriorly; posterior margin concave at middle, forming two semiovate plates with long setae. Ductus bursae weakly sclerotized entirely. Corpus bursae membranous, irregularly rounded.

#### Distribution.

Thailand.

#### Etymology.

The specific name is derived from the Latin adjective *triangulus* (triangular), referring to the shape of the distal half of the valva.

### Meleonoma dorsolobulata

Taxon classificationAnimaliaLepidopteraCosmopterigidae

Wang sp. n.

http://zoobank.org/5C1EA723-9160-4734-97B6-5DED4190C4E9

Figs 2 , 7

#### Type material.

Holotype: ♂, Thailand: Loei Province, Phu Luang Wildlife Sanctuary, 8–14.x.1984, ca. 700–800 m, leg. Karsholt, Lomholdt & Nielsen, genitalia slide No. ZMUC-NK047. Paratype: 1 ♂, same data as holotype.

#### Diagnosis.

This new species can be separated easily from its congeners by the sacculus having a lobate process at base dorsally in the male genitalia.

#### Description.

Adult (Fig. 2): Wing expanse 9.0–10.0 mm. Head yellow, tinged with greyish brown on vertex. Labial palpus yellow, with wide irregular brown rings at middle and at apex of second segment, as well as at 2/3 of third segment. Antenna yellow, dorsal surface black on scape, ringed with brown on flagellum. Thorax and tegula brown. Forewing yellow, with brown scales throughout, concentrated along costal 2/3, forming a narrow streak along basal 1/3, and forming a spot at middle; large blackish distal blotch from distal 1/5 of costal margin obliquely inward to end of fold; cell with small black spot at base and at middle, the former indistinct, with two black dots at end of cell, placed one above another, the lower one merged with large distal blotch, but distinct; cilia blackish brown. Hindwing and cilia grey. Legs whitish yellow; tibiae and tarsi greyish brown on outer surface, tibiae yellow at middle and at apex, tarsi yellow at apex of each segment.

Male genitalia (Fig. 7): Uncus nearly as long as saccus, basal half evenly wide, distal half gradually narrowed to blunt apex. Valva evenly wide from base to approximately 3/4, distal 1/4 slightly narrowed to rounded apex, upturned; costa concave medially; ventral margin straight basally, arched outward distally. Sacculus broad, subtriangular, apically produced to a hairy papillary process; large process arising from base of its dorsal margin, lobate, narrow basally, ovally inflated distally. Saccus triangular, wide at base, narrowed to apex. Juxta thin, broad V shaped. Aedeagus shorter than valva, with dense microspines on inner surface in distal 2/5, with a club-shaped process distally, approximately 1/3 length of aedeagus.

Female unknown.

#### Distribution.

Thailand.

#### Etymology.

The specific name, an adjective, is derived from the Latin *dorso*- (dorsal) and *lobulatus* (lobate), referring to the process at base of the dorsal margin of the sacculus.

### Meleonoma elongata

Taxon classificationAnimaliaLepidopteraCosmopterigidae

Wang sp. n.

http://zoobank.org/DB2F466F-73DE-4DE8-9E67-A26581717799

Figs 3 , 8

#### Type material.

Holotype: ♂, Thailand: Chieng Mai Province, Doi Inthanon Nat. Park, Siriphum, 21–24.X.1984. ca. 1200–1300 m, leg. Karsholt, Lomholdt & Nielsen, genitalia slide No. ZMUC-NK053.

#### Diagnosis.

This new species can be distinguished from its congeners in the male genitalia by having a narrowly elongate triangular sacculus and a broad saccus with an apical mastoid process.

#### Description.

Adult (Fig. 3). Wing expanse 10.0 mm. Head pale yellow, tinged with brown. Labial palpus pale yellow, with brown scales at middle and at apex of second segment, as well as at 2/3 of third segment. Antenna with dorsal surface black on scape, ringed with brown on flagellum. Thorax and tegula greyish brown, tinged with yellow. Forewing yellow, with brown scales throughout, denser along costal margin, forming large diffused spot at base and at middle; apex blackish brown; cell with small black spot at middle and at end; fold with small black spot at middle; cilia whitish yellow, tinged with brown at tornus. Hindwing and cilia pale grey. Legs pale yellow, with brown scales.

Male genitalia (Fig. 8). Uncus nearly as long as saccus, slightly wide at base, straight, stick-like. Valva somewhat knifelike in shape, widened medially, narrowed to base and apex; apex narrowly rounded, costa straight, ventral margin protruding outward medially. Sacculus less than half length of valva, elongate triangular; basal half broad, sclerotized dorsally and ventrally; distal half distinctly narrowed, apex narrowly rounded. Saccus broad, triangular, apically produced to a small process. Juxta a small ring. Aedeagus shorter than valva, slender, produced to a club-shaped process distally, with two slender, sclerotized, curved clubs distally.

Female unknown.

#### Distribution.

Thailand.

#### Etymology.

The specific name is derived from the Latin adjective *elongatus* (elongate), referring to the shape of the sacculus.

### Meleonoma bilobata

Taxon classificationAnimaliaLepidopteraCosmopterigidae

Wang sp. n.

http://zoobank.org/849C7976-09F3-4C20-A3CA-4A25E632306C

Figs 4 , 9

#### Type material.

Holotype: ♂, Thailand: Chieng Mai Province, 325 m, 15-30.x.1984, leg. Karsholt, Lomholdt & Nielsen, genitalia slide No. ZMUC-NK046.

#### Diagnosis.

This new species can be distinguished from its congeners in the male genitalia by the sacculus being apically bilobed.

#### Description.

Adult (Fig. 4). Wing expanse 9.0–10.0 mm. Head yellow. Labial palpus yellow. Antenna pale yellow, without distinct dark rings. Thorax and tegula yellow, tegula with brown scales at base. Forewing yellow, with brown and ochreous brown scales, with denser brown scales along costal margin, with denser pale ochreous brown scale along ventral margin; costal margin with a dark brown spot at base, with a dark brown diffusion beyond middle; large distal blotch black, from end of fold obliquely outward to apex; cell with small black spot at middle and at end of cell; fold with a small black dot at middle; cilia blackish brown. Hindwing and cilia grey. Legs whitish yellow; fore and mid legs with tibiae and tarsi blackish brown on outer surface, hind leg greyish brown; tarsi yellowish white at apex of each segment.

Male genitalia (Fig. 9). Uncus approximately twice as long as saccus, wide and triangular basally, gradually narrowed to pointed apex. Valva evenly wide except slightly narrowed at base, apex blunt; ventral margin with clustered long setae at 1/3. Sacculus wider than valva at base, shorter than saccus, apically concave at middle, forming two lobes: dorsal lobe longer, fingerlike, ventral lobe a rounded process. Saccus broad triangular, length same as height of tegumen. Aedeagus approximately 2/3 length of valva, strong and straight, with several tiny teeth along dorsal 1/6; cornutus a tiny spine, originating from a large sclerotized rectangular plate.

Female unknown.

#### Distribution.

Thailand.

#### Etymology.

The specific name is derived from the Latin adjective *bilobatus* (bilobate), referring to the apically bilobate sacculus.

### Meleonoma facialis

Taxon classificationAnimaliaLepidopteraCosmopterigidae

Li & Wang, 2002

Figs 5 , 10

Meleonoma facialis Li & Wang, 2002: 230.

#### Redescription.

Adult (Fig. 5): Wing expanse 10.0‒10.5 mm. Head pale yellow, with appressed scales. Labial palpus and antenna pale yellow, antenna ringed with pale brown on flagellum. Forewing yellow, with scattered black scales; black scales becoming denser at apex, forming irregular obscure blotch; costal margin with indistinct black spot at middle; small black dot set at middle of cell and at 2/3 of fold respectively; cilia same color as forewing. Hindwing and cilia greyish white. Legs yellowish white, fore and mid tibiae and tarsi brown on outer surface, with pale spots; mid tibia and tarsus tinged with brown scales.

Male genitalia (Fig. 10): Uncus somewhat conic. Valva narrow at base, slightly widened distally, apex rounded; transtilla distally rounded. Sacculus rectangular basally, with three processes distally: dorsal process longest, somewhat elliptically dilated distally; median process small pine-like; ventral process subtriangular, wide at base, narrowed gradually to 2/3, distal 1/3 sharply narrowed, uniform, apex bluntly rounded. Saccus large, triangular, apex roundly pointed. Aedeagus shorter than valva, straight, with one short sclerotized cornutus.

#### Material examined.

1♂, Thailand: Loei province, Phu Luang Wildlife Sanctuary, 8–14.x.1984, leg. Karsholt, Lomholdt & Nielsen, genitalia slide No. ZMUC-NK054.

#### Distribution.

Thailand, China (Jiangxi, Sichuan, Shaanxi, Yunnan).

## Supplementary Material

XML Treatment for Meleonoma

XML Treatment for Meleonoma triangula

XML Treatment for Meleonoma dorsolobulata

XML Treatment for Meleonoma elongata

XML Treatment for Meleonoma bilobata

XML Treatment for Meleonoma facialis

## References

[B1] Clarke JFG (1965) Catalogue of the type specimens of Microlepidoptera in the British Museum (Natural History) described by Edward Meyrick. Trustees of the British Museum (Natural History), London 5: 1−581.

[B2] Common FB (1996) Oecophoridae. In: Nielsen ES Edwards ED Rangsi TV (Eds) Checklist of the Lepidoptera of Australia. Monographs on Australian Lepidoptera 4 CSIRO, Collingwood, 59−89.

[B3] Edwards ED Nielsen BS (1996) Cosmopterigidae. In: Nielsen ES Edwards ED Rangsi TV (Eds) Checklist of the Lepidoptera of Australia. Monographs on Australian Lepidoptera 4 CSIRO, Collingwood, 102−106.

[B4] Gaede M (1939) Oecophoridae. Lepidopterorum Catalogus 92: 209−476.

[B5] Heikkila M Mutanen M Kekkonen M Kaila L (2014) Morphology reinforces proposed molecular phylogenetic affinities: a revised classification for Gelechioidea (Lepidoptera). Cladistics 30: 563–589. doi: 10.1111/cla.12064 10.1111/cla.12064 34794251

[B6] Meyrick E (1914) Exotic Microlepidoptera. Wilts, Marlborough, 1(8): 225−256.

[B7] Li HH (2002) The Gelechiidae of China (I) (Lepidoptera, Gelechiidae). Nankai University Press, Tianjin, 538 pp [In Chinese]

[B8] Li HH Wang SX (2002) A study on the genus *Meleonoma* Meyrick from China, with descriptions of two new species (Lepidoptera: Cosmopterigidae). Acta Entomologica Sinica 45(2): 230‒233.

[B9] Li HH Wang XP (2004) New Species of *Meleonoma* Meyrick (Lepidoptera: Cosmopterigidae) from China. Entomotaxonomia 26(1): 35−40.

[B10] Lvovsky AL (2010) A new genus of broad-winged moths of the family Cryptolechiidae (*Acryptolechia*, Lepidoptera, Gelechioidea) from southeastern Asia. Zoologicheskii Zhurnal 89(3): 378‒381. doi: 10.1134/s0013873810020119

[B11] Lvovsky AL (2015) Composition of the subfamily Periacminae (Lepidoptera, Lypusidae) with descriptions of new and little known species of the genus *Meleonoma* Meyrick, 1914 from south, east, and south-east Asia. Entomological Review 95(6): 766–778. doi: 10.1134/S0013873815060111

[B12] Viette PEL (1955) Nouveaux Tineoidea (s.l.) de Madagascar (Lep.). Annales de la Société Entomologique de France 123: 75−114.

